# Computed tomography-guided lung biopsy with rapid on-site evaluation for diagnosis of lung lesions: a meta-analysis

**DOI:** 10.1186/s13019-023-02212-6

**Published:** 2023-04-10

**Authors:** Di Wu, Yue-Yue Liu, Tao Wang, Ya-Yong Huang, Ping Xia

**Affiliations:** 1 Department of Pathology, Xuzhou Central Hospital, Xuzhou, China; 2 Department of Radiology, Xuzhou Central Hospital, Xuzhou, China

**Keywords:** Lung biopsy, Rapid on-site evaluation, Meta-analysis

## Abstract

**Background:**

Lung biopsy (LB) procedures performed with computed tomography (CT guidance can enable the reliable diagnosis of lung lesions. These diagnostic efforts can be further expedited through a rapid on-site evaluation (ROSE) approach, allowing for the rapid assessment of collected tissue samples to gauge the adequacy of these samples, their features, and associated cytomorphological characteristics. The present analysis was developed to examine the safety and efficacy of CT-guided LB with ROSE as a means of diagnosing lung lesions.

**Methods:**

Studies published as of July 31, 2022 in the PubMed, Embase, and Wanfang databases were identified for this meta-analysis. Diagnostic accuracy was the primary endpoint, while secondary endpoints included the operative duration, the number of punctures, and rates of lung hemorrhage, pneumothorax, and secondary LB.

**Results:**

This meta-analysis included 6 total studies. Relative to CT alone, CT with ROSE was associated with a significant increase in diagnostic accuracy (*P* < 0.00001). In contrast, there were no significant differences between these two groups with respect to the operative duration (*P* = 0.86), the number of punctures (*P* = 0.60), or the rates of pneumothorax (*P* = 0.82) or lung hemorrhage (*P* = 0.81). Pooled secondary LB rates were significantly lower for patients that underwent CT with ROSE relative to patients in the CT only group (*P* = 0.0008). Significant heterogeneity was detected for the operative duration (*I*^2^ = 94%) and number of punctures (*I*^2^ = 98%) endpoints, while no publication bias was detected for any study endpoints.

**Conclusions:**

These results suggest that ROSE may contribute to significant improvements in the diagnostic accuracy of CT-guided LB without contributing to higher rates of complications.

## Introduction

Lung biopsy (LB) is a standard approach that is used for the safe and reliable diagnosis of nodules and masses present within the lungs [1–3]. Prior work suggests that the actual diagnostic accuracy of LB procedures can range from 65 to 94% [4–7], with a range of factors including the lesion size, needle type (core vs. fine needle), and imaging guidance technique (bronchoscopy, computed tomography [CT], or CT fluoroscopy) all impacting these diagnostic yields [5–8]. A failure to obtain an adequate biopsy specimen has been suggested to be an important cause of the misdiagnosis of lung malignancies in some reports [8, 9].

Rapid on-site evaluation (ROSE) offers an approach to rapidly conduct cytomorphological characteristics of tissues obtained from biopsy procedures in order to gauge their adequacy and malignancy. ROSE procedures performed by experienced pathologists can contribute to improved LB diagnostic accuracy [10]. ROSE techniques have been frequently employed in the context of bronchoscopy-guided biopsy procedures [10–15]. In contrast, there have been fewer studies regarding the application of ROSE approaches in the context of CT-guided LB [16–22]. As the results from an individual have the potential to be subject to bias derived from many sources, meta-analyses are warranted to mitigate such bias and to improve the overall statistical power of associated results [23].

Accordingly, the present study was conducted with the goal of examining the safety and diagnostic efficacy of combining CT-guided LB and ROSE approaches when evaluating lung lesions.

## Materials and methods

### Study selection

This meta-analysis was conducted based on Preferred Reporting Items for Systematic Reviews and Meta-Analyses (PRISMA) guidelines [24]. All studies published as of July 31, 2022 in the PubMed, Embase, and Wanfang databases were identified with the following search strategy: ((((Computed Tomography) OR (CT)) AND ((lung) OR (pulmonary))) AND (biopsy)) AND ((Rapid On-Site Evaluation) OR (ROSE)). This meta-analysis was registered at INPLASY.COM (No. INPLASY202280063).

Inclusion criteria:

- Types of studies: comparative studies;
- Diseases: lung lesions necessitating CT-guided LB;
- Types of interventions: CT-guided LB with ROSE versus CT-guided LB only;
- Languages: no limitations.

Exclusion criteria:

- single-arm studies;
- duplicate studies;
- non-human studies;
- case reports, letters, and reviews.

### Data extraction and quality assessment

Two researchers (D.W. and T.W.) independently extracted baseline study data (first author, country, year of publication, study design, and quality scores), baseline patient data (number of patients, gender ratio, patient age, lesion diameter, lesion-pleura distance, and final diagnosis), and outcome data (diagnostic accuracy, number of punctures, operative duration, complications, and secondary LB rates). Discrepancies were resolved by a third investigator (YY. L.).

The Cochrane risk of bias tool was used to examine the quality of randomized controlled trials (RCTs) based on random sequence generation, allocation concealment, participant and personnel blinding, outcome assessment blinding, selective reporting, incomplete data, and other forms of bias.

Non-RCT quality was assessed using the Newcastle–Ottawa scale (NOS), consisting of criteria pertaining to selection (4 points), comparability (2 points), and outcomes (3 points) [25]. High-quality studies were those with a NOS score ≥ 7.

### Endpoints

Diagnostic accuracy was the primary endpoint for this study, and was considered positive if the biopsy-based diagnosis was consistent with the final pathological diagnosis. Secondary study endpoints included operative duration, numbers of punctures, and the rates of pneumothorax, lung hemorrhage, and secondary LB. The secondary LB was conducted if the primary LB failed to obtain sufficient sample for the pathological diagnosis [17]. The operation duration was defined as the time from patients lying on the CT bed to getting out of the CT bed. In ROSE group, the operation time contained the CT-guided LB and ROSE time.

### Meta-analysis

RevMan v5.3 was used to conduct pooled analyses. Continuous variables were compared using mean difference (MD) values and 95% confidence intervals (CIs), while categorical variables were compared based on pooled odds ratios (ORs) and 95% CIs. Heterogeneity was analyzed with the *I*^2^ statistic and the Q test, with significant heterogeneity being denoted by an *I*^2^ > 50%. Random-effects models were used in the context of significant heterogeneity, while fixed-effects models were otherwise conducted. A leave-one-out approach was used to perform sensitivity analyses aimed at determining the sources of any heterogeneity. Egger’s test was used to detect possible publication in Stata v12.0, and *P* < 0.05 was established as the threshold of significance.

## Results

### Study selection

**Fig. 1:**
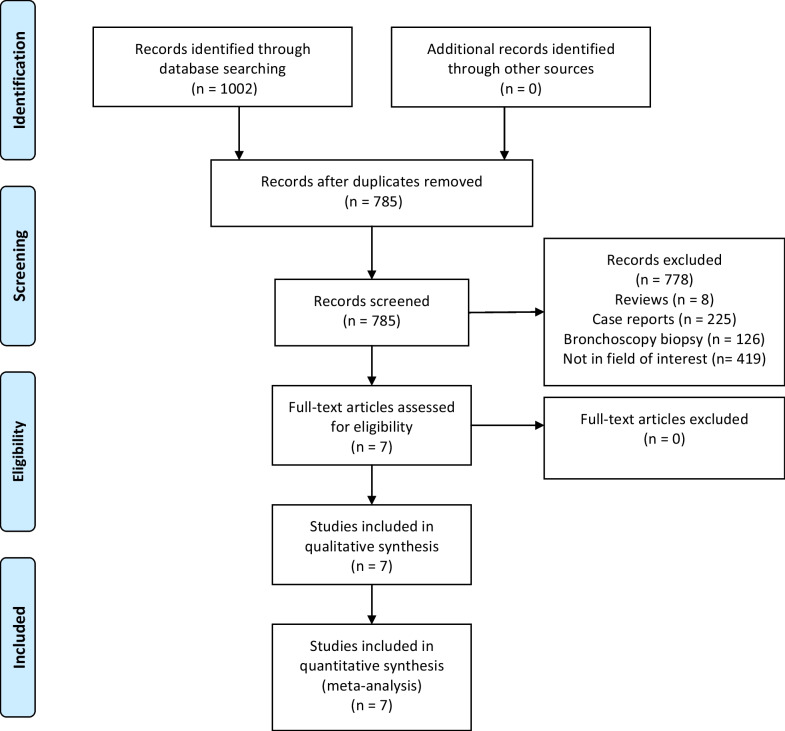
Flowchart diagram of our meta-analysis

The initial search strategy yielded 1,002 studies, of which 785 were retained following the removal of duplicate entries. In total, 7 studies were incorporated into the final meta-analysis [16–22]. For further details regarding the study selection process, see Fig. 1.

**Fig. 2:**
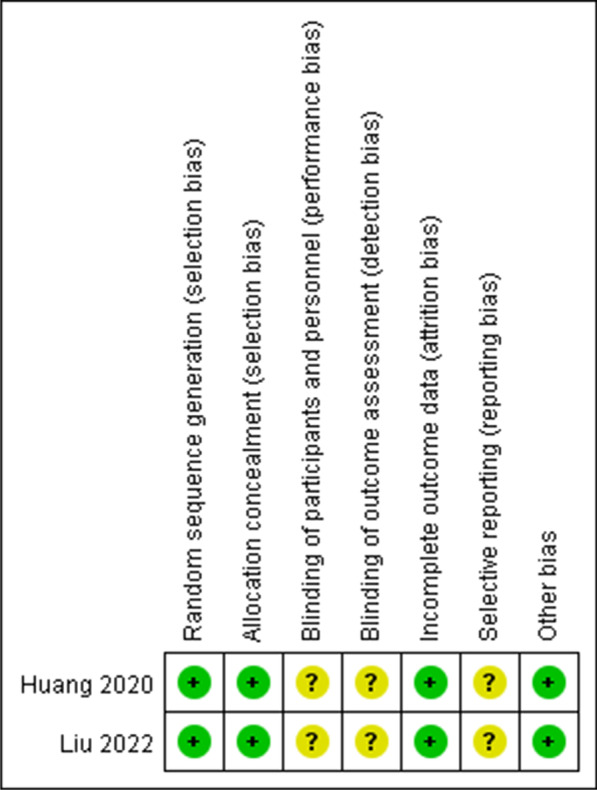
Cochrane risk-of-bias tool for the included RCTs

Of these 7 studies, 2 were RCTs [16, 18] while 5 were retrospective in nature [17, 19–22]. Both RCTs exhibited an unclear risk of bias with respect to performance, detection, and reporting (Fig. 2). NOS scores for the included retrospective analyses ranged from 7–8. Core needles were used for LB procedures in 4 studies [17, 20–22], while 1 used a fine needle biopsy approach [19], and two studies did not specify the needle type(s) used [16, 18] (Table 1).

**Table 1:** Baseline data of the studies

	First author	Year	Country	Design	Puncture needle types	NOS
1	Huang [16]	2020	China	RCT	Not provided	–
2	Li [17]	2020	China	Retrospective	Core needle	8
3	Liu [18]	2022	China	RCT	Not provided	–
4	Peng [19]	2020	China	Retrospective	Fine needle	7
5	Wang [20]	2021	China	Retrospective	Core needle	8
6	Yiminniyaze [21]	2022	China	Retrospective	Core needle	8
7	Zhang [22]	2021	China	Retrospective	Core needle	8

NOS, Newcastle-Ottawa Scale; RCT, randomized controlled trial

These studies included 748 and 673 patients who respectively underwent CT-guided LB procedures with and without ROSE (Table 2). Baseline data were comparable between these two patient groups in all studies.

**Table 2:** Baseline data of the patients in these studies

Author	Groups	Patients (n)	Mean age	Gender (M/F)	Mean diameter	Mean lesion-pleura distance	Final diagnoses (malignant/benign)
Huang [16]	CT + ROSE	53	64.5 y	30/23	Not given	Not given	49/4
	CT alone	54	64.4 y	33/21	Not given	Not given	Not given
Li [17]	CT + ROSE	58	59.8 y	30/28	1.4 cm	4.5 cm	32/26
	CT alone	50	59.3 y	28/22	1.3 cm	4.6 cm	28/22
Liu [18]	CT + ROSE	56	59.8 y	30/26	2.5 cm	4.4 cm	32/24
	CT alone	52	59.4 y	28/24	3.4 cm	4.7 cm	28/24
Peng [19]	CT + ROSE	132	57.3 y	86/46	Not given	Not given	61/71
	CT alone	102	56.4 y	66/36	Not given	Not given	54/48
Wang [20]	CT + ROSE	148	59.8 y	98/50	2.9 cm	4.2 cm	92/56
	CT alone	143	59.7 y	94/49	2.9 cm	4.0 cm	Not given
Yiminniyaze [21]	CT + ROSE	163	63 y	108/55	< / ≥ 3 cm: 36/127	Not given	157/6
	CT alone	122	64.5 y	85/37	</≥ 3 cm: 23/99	Not given	113/9
Zhang [22]	CT + ROSE	138	59.9 y	79/59	11.1 cm	Not given	95/43
	CT alone	150	60.1	96/54	10.9 cm	Not given	92/58

CT, computed tomography; M, male; F, female; ROSE, rapid on-site evaluation

### Diagnostic accuracy

**Fig. 3:**
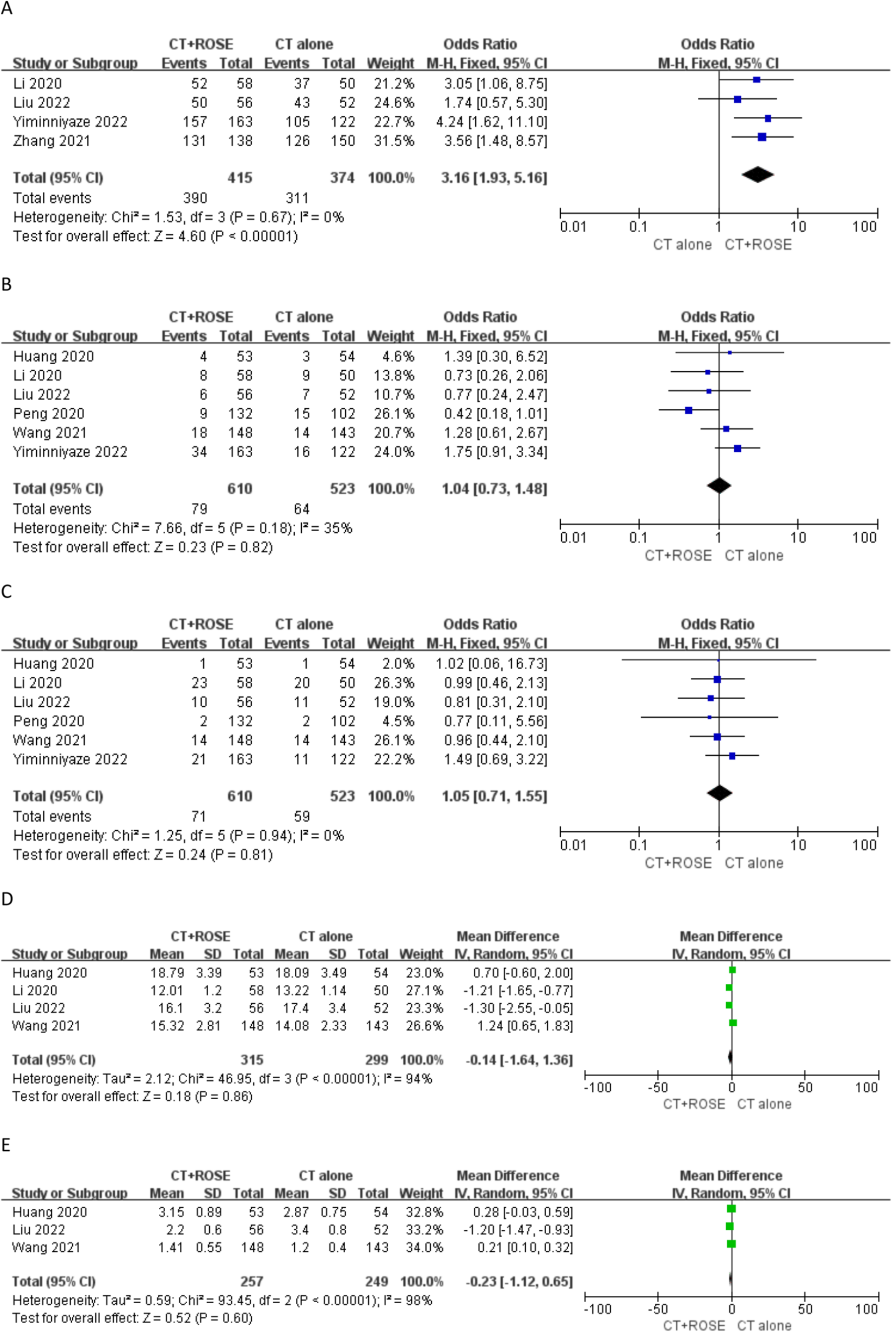

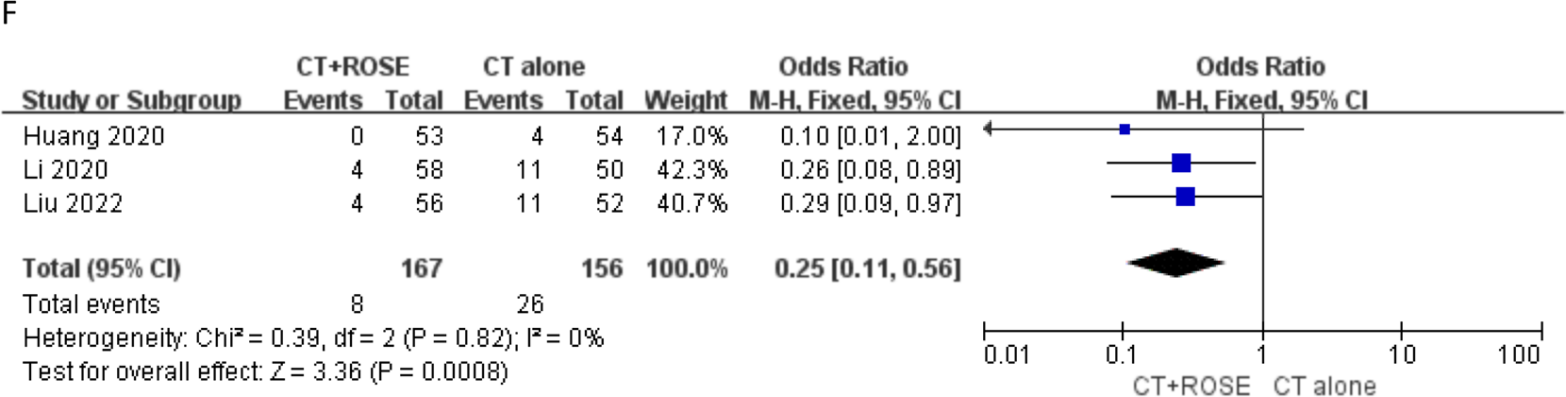
**a** The pooled diagnostic accuracy rate was significantly higher in CT-guided LB with ROSE group than that in CT-guided LB alone group; **b** The pooled pneumothorax rates were comparable between 2 groups; **c** The pooled lung hemorrhage rates were comparable between 2 groups; **d** The pooled operative time was comparable between 2 groups; **e** The pooled numbers of puncture were comparable between 2 groups; and **f** The pooled secondary LB rate was significantly lower in CT-guided LB with ROSE group than that in CT-guided LB alone group

In total, 4 studies reported diagnostic accuracy rates [17, 18, 21, 22]. These pooled diagnostic accuracy rates were significantly higher for patients who underwent CT-guided LB with ROSE relative to those who underwent CT-guided LB alone (94.0% vs. 83.2%, OR: 3.16, *P* < 0.00001, Fig. 3a). No significant heterogeneity was detected (*I*^2^ = 0%), and Egger’s test revealed no evidence of publication bias (*P* = 0.243) (Table 3).

**Table 3:** Subgroup analyses based on the use of core needle

	Number of studies	OR/MD (95% CI)	Heterogeneity (%)	Favor
Diagnostic accuracy	3	3.62 (2.09, 6.29), *P* < 0.0001	*I*^2^ = 0	CT + ROSE
Pneumothorax	3	1.34 (0.87, 2.07), *P* = 0.19	*I*^2^ = 0	–
Lung hemorrhage	3	1.13 (0.72, 1.76), *P* = 0.59	*I*^2^ = 0	–
Operative time	2	0.01 (− 2.39, 2.41), *P* = 1.00	*I*^2^ = 99	–

OR, odds ratio; MD, mean difference; ROSE, rapid on-site evaluation; NA, not applicable

### Pneumothorax

Pneumothorax rates were provided in 6 studies [16–21], and these rates did not differ significantly between the two study groups (13.0% vs. 12.2%, OR: 1.04, *P* = 0.82, Fig. 3b). No significant heterogeneity was detected for this endpoint (*I*^2^ = 35%), and Egger’s test revealed no evidence of publication bias (*P* = 0.441).

### Lung hemorrhage

Lung hemorrhage rates were provided in 6 studies [16–21], and these rates did not differ significantly between the two study groups (11.6% vs. 11.3%, OR: 1.05, *P* = 0.81, Fig. 3c). No significant heterogeneity was detected for this endpoint (*I*^2^ = 0%), and Egger’s test revealed no evidence of publication bias (*P* = 0.57).

### Operative duration

Four studies reported data pertaining to operative duration [16–18, 20]. No significant differences in these pooled operative duration values were observed when comparing study groups (MD: -0.14, *P* = 0.86, Fig. 3d). Significant heterogeneity was detected for this endpoint (*I*^2^ = 94%), but sensitivity analyses failed to establish the source of this heterogeneity. Egger’s test revealed no evidence of publication bias (*P* = 0.181).

### Number of punctures

Three studies provided data regarding the number of punctures [16, 18, 20], with no significant differences in this number between study groups in pooled analyses (MD: − 0.23, *P* = 0.60, Fig. 3e). Significant heterogeneity was detected for this endpoint (*I*^2^ = 98%), and the study performed by Liu et al. [18] was identified as the source of this heterogeneity in a sensitivity analysis. study. Egger’s test revealed no evidence of publication bias (*P* = 0.455).

### Secondary LB rates

Secondary LB rates were reported in 3 studies [16–18]. A significantly lower secondary LB rate was evident for patients who underwent CT-guided LB with ROSE relative to patients who underwent CT-guided LB alone (4.8% vs. 16.7%, OR 0.25, *P* = 0.0008, Fig. 3f). No significant heterogeneity was detected (*I*^2^ = 0%), and Egger’s test revealed no evidence of publication bias (*P* = 0.102).

### Subgroup analyses

Subgroup analyses were performed for patients that underwent core needle biopsy procedures. Data regarding diagnostic accuracy rates, operative duration, and the rates of pneumothorax and lung hemorrhage were successfully pooled for this analysis. Significantly higher pooled diagnostic accuracy rates were observed for patients who underwent ROSE relative to those who did not (*P* < 0.0001). In contrast, similar pooled pneumothorax rates (*P* = 0.19), lung hemorrhage rates (*P* = 0.59), and operative time (*P* = 1.00) were evident in these study groups.

## Discussion

In this meta-analysis, diagnostic efficacy and safety outcomes were compared for the CT-guided LB of lung lesions with or without the incorporation of a ROSE approach. Overall, these pooled analyses revealed that ROSE contributed to significant improvements in CT-guided LB diagnostic accuracy without prolonging the operative duration or increasing rates of procedure-related complications as compared to CT-guided LB alone.

Diagnostic accuracy is the most important outcome in studies analyzing CT-guided LB approaches [2, 26, 27]. In the present report, ROSE was found to significantly improve these diagnostic rates by approximately 10.8% relative to CT-guided LB alone, while also significantly decreasing the rates of secondary LB in evaluated patients. This is consistent with the ability of ROSE to provide rapid insight regarding the cytomorphological adequacy and other characteristics of LB samples such that these ROSE-based preliminary diagnoses can be used to guide subsequent patient management [18].

ROSE-based diagnoses were highly consistent with final pathological diagnoses, with accurate rates ranging from 89.3 to 95.7% [18, 21]. As ROSE relies on the rapid staining of cell smears, however, it is not sufficient as a final diagnostic tool given that it fails to provide any histological or morphological information and cannot differentiate between lung cancer pathological subtypes, instead only allowing clinicians to judge whether a given lung lesion is malignant or benign [18].

The most common complications associated with CT-guided LB procedures include lung hemorrhage and pneumothorax. In the present analysis, no differences in the rates of either of these complications were observed when comparing CT-guided LB procedures performed with and without ROSE. As no significant heterogeneity was observed for these endpoints, this also suggests that these results are stable. Prior research has suggested that factors that do impact the rates of these CT-guided LB-related complications include emphysema, small lesions, non-prone positioning, a longer lesion-pleura distance, and a greater number of needle pathways [4, 21, 26, 28]. No significant differences in the pooled number of punctures were observed when comparing these two groups, potentially explaining why no reduction in safety for the CT-guided LB procedure was observed with ROSE incorporation.

Operative duration was comparable in both groups. While the ROSE procedure does require some time to complete the requisite dying and associated analyses, operator proficiency can effectively limit this time such that no significant differences in operative duration were observed with the integration of ROSE into the LB workflow. However, this endpoint was subject to significant heterogeneity. The significant heterogeneity may be subject to bias in retrospective studies, and different operators’ skill and experence. Further well designed prospective studies should be conducted to vadilate this result.

Core needle use can achieve greater levels of sample adequacy relative to fine needle use [6]. Accordingly, subgroup analyses for core needle biopsy procedures were performed, revealing that ROSE significantly improved CT-guided core needle biopsy diagnostic accuracy without any adverse safety-related outcomes.

There are certain limitations to this meta-analysis. For one, the majority of the included studies were retrospective in nature and thus subject to a high risk of bias. In addition, the majority of these studies incorporated several lung lesion types, including both lung masses and lung nodules. Moreover, one study did not specify the needle type used for biopsy procedures, which is an important consideration given that needle type can impact both diagnostic accuracy and complication rates. This may have impacted the results of needle type-based subgroup analyses performed herein. Fourth, all included studies were conducted in China, and additional meta-analyses should thus aim to incorporate data derived from other clinical research centers throughout the world.

## Conclusion

In summary, these results suggest that the incorporation of ROSE procedures into the LB workflow may significantly improve CT-guided LB diagnostic accuracy without compromising the safety of this approach.

## Abbreviations

CT: Computed tomography
LB: Ung biopsy
NOS: Newcastle-Ottawa scale
RCT: Randomized controlled trial
ROSE: Rapid on-site evaluation
